# Insights into the role of gut microbiota in obesity: pathogenesis, mechanisms, and therapeutic perspectives

**DOI:** 10.1007/s13238-018-0546-3

**Published:** 2018-05-03

**Authors:** Lijuan Sun, Lanjing Ma, Yubo Ma, Faming Zhang, Changhai Zhao, Yongzhan Nie

**Affiliations:** 1 State Key Laboratory of Cancer Biology & Institute of Digestive Diseases Xijing Hospital, The Fourth Military Medical University, Xi’an, 710032 China; 2 Department of Clinical Nutrition, Xi Jing Hospital, The Fourth Military Medical University, Xi’an, 710032 China; 3 Department of Dermatology and Venereology, Peking University First Hospital, Research Center for Medical Mycology, Peking University, Beijing, 100034 China; 4 Medical Center for Digestive Diseases, The Second Affiliated Hospital of Nanjing Medical University, Nanjing, 210001 China

Concern about health risks associated with rising obesity has become nearly universal, with the mean body mass index (BMI) and the prevalence of obese and overweight individuals increasing substantially worldwide during the previous three decades. Unfortunately, prevention and treatment of obesity and related complications have proven complex, and successful strategies to tackle this pathology remain limited. Epidemiological studies have highlighted potential environmental exposures, including diet, energy expenditure, early life influences, sleep deprivation, endocrine disruptors, chronic inflammation, and microbiome status, contributing to higher risk of obesity (Franks and McCarthy, 2016). Among these, the microbiome has received extensive attention during the previous decade.

Variation in gut microorganisms might play an important role in the pathogenesis of obesity. Although the composition of intestinal microbiota is highly diverse in healthy individuals, those exhibiting overall adiposity, insulin resistance and dyslipidemia are characterized by low bacterial richness (Le Chatelier et al., 2013). Moreover, composition of gut microbiota in obesity individuals differs from that in lean individuals, although inconsistent changes have been reported. *Bacteroidetes* prevalence is lower in obese people, with this proportion increasing along with weight loss based on a low-calorie diet (Ley et al., 2006a). *Lactobacillus* and *Clostridium* species are associated with insulin resistance, with *Lactobacillus* positively correlated with fasting glucose and HbA1c levels, whereas *Clostridium* showed a negative correlation with these parameters (Karlsson et al., 2013). These data suggest that specific bacterial phyla, class, or species or bacterial metabolic activities could be beneficial or detrimental to the onset of obesity. Therefore, the gut microbiome has been suggested as a driving force in the pathogenesis of obesity.

Causal evidence linking intestinal microbiota to obesity mostly originates from animal studies. Germ free (GF) mice are resistant to high-fat diet (HFD)-induced obesity, despite a higher food intake. Interestingly, administration of subtherapeutic antibiotic therapy increased adiposity and metabolism-related hormone levels in young mice, with these changes altering the copies of key genes involved in the metabolism of carbohydrates to short-chain fatty acids (SCFAs) and the regulation of hepatic metabolism of lipids and cholesterol (Cho et al., 2012). Furthermore, colonization of GF mice with “obese microbiota” resulted in a significantly greater increase in total body fat than colonization with “lean microbiota” (Turnbaugh et al., 2006). Notably, GF mice that received fecal microbiota transplantation (FMT) from an obese donor gained more weight as compared with those receiving it from a lean donor (Ridaura et al., 2013), with this result further accelerating the establishment of the causal role of gut microbiota in the development of obesity.

Mechanisms by which gut microbiota promote metabolic disturbances are not well understood. To date, leading theories about the mechanisms include changes in molecular signaling chemicals released by bacteria in contact with local tissue or distant organs (Schroeder and Backhed, 2016; Meijnikman et al., 2017) (Fig. 1).

**Figure 1:**
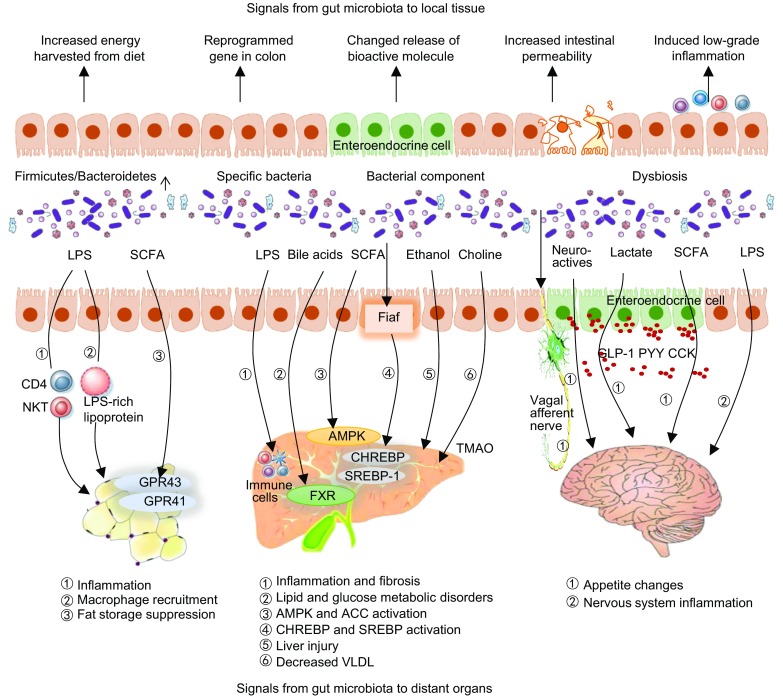
Impact of gut microbiota on local and distant organs contributes to obesity development and progression. In local tissues, obesity-associated gut microbiota have an increased capacity to harvest energy from the diet, stimulate gene reprogramming in the colon, change polypeptide hormones and other bioactive molecules released by EC cells, decrease the intestinal barrier, and disturb immune homeostasis. Gut microbiota also communicate with host adipose tissue and the liver and brain. Microbiota-fat-signaling axis. Gut microbiota participates in the regulation of adipogenesis through distinct mechanisms. LPS triggers an immune response along with inflammation and immune-cell infiltration. SCFAs also participate in insulin-mediated fat accumulation in adipocytes via activation their receptors GPR43 and GPR41, which inhibits lipolysis and encourages adipocyte differentiation. Gut-liver axis. The presence of a dysbiotic microbiome causes subsequent increases in gut permeability to bacteria-derived pathogens, including LPS and ethanol. In the liver, LPS causes inflammation by stimulating immune cells. Certain metabolites, such as bile acids, SCFAs, and TMAO, also play a role in NAFLD pathophysiology. Microbiota-brain-gut axis. Gut afferent neuron and gut hormones are key signaling molecules involved in gut-brain communication and host metabolism. Bioactive molecules involved in this process include LPS, gut peptides, SCFAs and lactate

Changes in gut microbiota perturb homeostatic interaction between microbiota and the intestine and might contribute to metabolic disorders. Local contacts between microbiota and intestine cells determine which signals are sensed and presented and which reactions are subsequently initiated. Increased energy harvesting by obesity associated gut microbiota is another possible explanation for obesity. The obese microbiome is typified by a reduced presence of taxa belonging to the *Bacteroidetes* phylum and a proportional increase in members of the *Firmicutes* phylum, revealing an association with a higher presence of enzymes for complex carbohydrate degradation and fermentation (Ley et al., 2006b), which are related to elevated levels of energy harvesting from the diet (Jumpertz et al., 2011). Additionally, the gut microbiome can stimulate reprogramming of gene expression in the colon (Qin et al., 2018). Fasting-induced adiposity factor (Fiaf; also known as angiopoietin-like protein 4), a circulating lipoprotein lipase inhibitor whose expression is normally selectively suppressed in the gut epithelium by microbiota (Backhed et al., 2007), plays a central role in triglyceride metabolism (Kim et al., 2010) by inhibiting lipoprotein lipase (LPL) production in adipose tissue and modulating fatty acid oxidation. Some specific components of microbiota might suppress Fiaf in the intestinal epithelia and potentially stimulate host weight gain by impairing triglyceride metabolism and promoting fat storage. Polypeptide hormones and other bioactive molecules released by enterochromaffin (EC) cells in the intestine are also involved in regulating food intake (Gribble and Reimann, 2016). Various Toll-like receptors (TLRs) expressed in EC cells recognize different pathogen-associated molecular patterns and alter the release of polypeptide hormones and other bioactive molecules. For example, lipopolysaccharide (LPS) molecules from Gram-negative bacteria and recognized by TLR4 cause secretion of cholecystokinin (CCK) through a mechanism dependent upon MyD88 and protein kinase C (Bogunovic et al., 2007; Palazzo et al., 2007). The altered intestinal barrier and subsequent translocation of bacteria or bacterial products is now regarded as an important mechanism associated with obesity. Exposure of cultured intestinal epithelial cells to commensal or probiotic microbial species results in upregulation and increased phosphorylation of key tight-junction proteins (Ewaschuk et al., 2008; Anderson et al., 2010). Additionally, some bacterial products play an important role in regulating the intestinal barrier, with associated SCFAs capable of differentially regulating prostaglandin production in myofibroblasts, thereby stimulating mucin-2 expression in intestinal epithelial cells (Willemsen et al., 2003). Obesity is related to the generation of low-grade, chronic inflammation (Lumeng and Saltiel, 2011), and gut-derived antigens are considered potential triggers for this activity. Furthermore, dysbiosis of microbiota can influence the innate and adaptive immune systems of the host via microbial cell components and metabolite signals.

Microbiota has effects beyond local tissue, with adipose tissue considered a primary target. Obesity is characterized as a massive expansion of adipose tissue, and growing evidence suggests that gut microbiota contribute to metabolic disorders through an axis of communication with adipose tissue. LPS has been identified as a triggering factor for insulin resistance in adipose tissue. In the trans-cellular pathway, LPS is actively transported into the cell in proportion to the fat content of the chime, followed by transfer to other lipoproteins by translocases. LPS-rich lipoproteins are absorbed by especially large adipocytes exhibiting high metabolic activity (Hersoug et al., 2016). Additionally, SCFAs produced by gut microbiota also participate in insulin-mediated fat accumulation in adipocytes through activation of the SCFA receptors G-protein coupled receptor (GPR)43 and GPR41 in adipocytes, which subsequently inhibits lipolysis and encourages adipocyte differentiation (Kimura et al., 2013). Intriguingly, MicroPET-CT results showed that microbiota depletion leads to increased glucose disposal primarily in inguinal subcutaneous adipose tissue and perigonadal visceral adipose tissue (Suarez-Zamorano et al., 2015), thereby stimulating energy expenditure through thermogenesis. This process was largely dependent upon eosinophils and the type 2 cytokines interleukin (IL)-4, IL-13, and IL-5 through alternative activation of M2 macrophages. Specific metabolic effects of some genes in adipocytes are also largely dependent upon altered microbiota composition. A recent study demonstrated that specific deletion of the endocannabinoid system synthesizing enzyme in adipocytes (NAPE-PLD) induced obesity and altered the browning program, with these changes partly mediated by a shift in gut-microbiota composition. These findings support those from a previous study showing that FMT was also capable of partially transferring a phenotype to GF mice (Geurts et al., 2015).

The liver is continually exposed to gut-derived signals, including those originating from bacterial components and products, through the receipt of ~70% of the blood supply from the portal vein, which enables direct venous outflow from the intestines. Alteration of gut commensal bacteria has consistently been associated with increased risk of obesity related liver disease [e.g., nonalcoholic fatty liver disease (NAFLD)], with a dysbiotic microbiome frequently observed among obese individuals with NAFLD (Turnbaugh et al., 2009). NAFLD severity is associated with gut dysbiosis and a shift in the metabolic function of gut microbiota, with *Bacteroides* abundance independently associated with nonalcoholic steatohepatitis (NASH), and *Ruminococcus* abundance associated with significant fibrosis (Boursier et al., 2016). GF mice colonized with intestinal bacteria from HFD mice develop NAFLD and had display hepatic lipid levels similar to those of donor mice, thereby implicating the gut microbiome in hepatic lipid accumulation (Le Roy et al., 2013).

Multiple lines of evidence link dysbiosis to obesity related liver disease. NAFLD presents with intestinal-bacterial overgrowth and enhanced intestinal permeability. Following bacterial generation of LPS, NF-κB is stimulated to recruit inflammatory cells, thereby promoting inflammation and fibrosis in advanced NAFLD (Elsharkawy and Mann, 2007). LPS also activates the NLRP3 infammasome via TLR4 and TLR9, which play an important role in fibrosis development in NAFLD (Wree et al., 2014). In addition to direct interactions associated with gut-derived bacterial signals, certain metabolites also play a role in NAFLD pathophysiology. Gut microbiota has profound effects on bile-acid metabolism by promoting deconjugation, dehydrogenation and dehydroxylation of primary bile acids. Additionally, alteration of the gut microbiome leads to changes in the bile-acid pool, which affects the farnesoid X receptor (FXR) nuclear antagonist involved in the regulation of bile acid, as well as lipid and glucose metabolism (Li et al., 2013), and could cause metabolic dysfunction, including obesity and insulin resistance. SCFAs lower hepatic fatty acid synthase activity and increase hepatic lipid oxidation, with this shift associated with increased phosphorylation and activation of adenosine monophosphate-activated protein kinase (AMPK) and its downstream target acetyl-CoA carboxylase (den Besten et al., 2015). Fiaf is also involved in the mechanism linking the microbiome to NAFLD, where dysbiotic microbiota inhibits Fiaf secretion from intestinal cells and leads to activation of LPL, carbohydrate-responsive element binding protein, (ChREBP) and sterol regulatory element-binding protein 1(SREBP-1), and subsequent triglyceride accumulation in the liver (Backhed et al., 2004). Ethanol is another bacterial product involved in NAFLD progression, with blood ethanol levels statistically significantly increased in patients with NASH (Zhu et al., 2013) and possibly related to a higher abundance of alcohol-producing *Proteobacteria*. Trimethylamine N-oxide (TMAO) is a small, colorless amine oxide generated from choline by gut-microbial metabolism, and its accumulation reduces bile-acid-synthetic enzymes (Cyp7a1 and Cyp27a1) and bile-acid transporters (Oatp1, Oatp4, Mrp2 and Ntcp) in the liver (Koeth et al., 2013). Additionally, patients with NAFLD have a higher level of *Erysipelotrichia*, which are linked to choline metabolism (Spencer et al., 2011). Therefore, dysbiosis in obesity is likely to impact metabolic homeostasis.

Similarly, the central nervous system receives constant neural and chemical input from the gut and is responsible for integrating this information and generating appropriate food-reward signaling to maintain homeostasis (Fetissov, 2017). Bacteria and their metabolites might target the brain directly via vagal stimulation or indirectly through immune-neuroendocrine mechanisms (Torres-Fuentes et al., 2017a). The vagal nerve transmits information from enteral content to the nucleus tractus solitaries, where the information is then distributed to the hypothalamus, which regulates appetite, food intake and energy balance. Activation of the vagus nerve is partly dependent upon the secretion of chemical signals, such as gut peptide YY (PYY), glucagon-like peptide 1 (GLP-1) and CCK, by enteroendocrine cells. Additionally, several bacterial strains can modify gut-hormone secretion (Balakumar et al., 2016), which can also be released into circulation and thereby affect appetite and satiety via hypothalamic neuroendocrine pathways. This effect is at least partly dependent upon microbiota-derived metabolites. For example, lactate is the preferred substrate for neurons and contributes to postprandial satiety. Moreover, lactate is capable of being abundantly produced in the gut by *Lactobacilli*, *Enterobacteriaceae* and *Bifidobacteria* (Silberbauer et al., 2000). SCFAs not only serve as an important energy source, but also act as chemical messengers or signaling molecules through their ability to increase proglucagon and pro-PYY gene expression to increase plasma GLP-1 and PYY levels and either inhibit ghrelin secretion (Nohr et al., 2013) or regulate appetite by releasing it into circulation. However, the reported results specific to this activity are inconsistent. For example, acetate, the main SFCA secreted by intestinal bacteria, is taken up by the brain and plays a direct role in suppressing appetite via central hypothalamic mechanisms (Frost et al., 2014). Another study reported that increased production of acetate by altered gut microbiota leads to activation of the parasympathetic nervous system accompanied by increased ghrelin secretion, hyperphagia and obesity (Perry et al., 2016). Furthermore, gut bacteria can also affect the central control of appetite by producing neuroactive metabolites, including serotonin and γ-aminobutyric acid, because these neurotransmitters are involved in the normal regulation of energy balance. Additionally, gut microbiota is associated with inflammation via LPS, which leads to activation of immune cells (B cells or dendritic cells) and cytokine production (Torres-Fuentes et al., 2017b).

Overall, two broad, but not mutually exclusive, mechanistic categories exist for the effects of microbiota on metabolic disorders: 1) direct interaction of gut microbiota with local tissue and 2) indirect interaction with distant organs through metabolic signals. It is tempting to speculate that the effects of microbiota on metabolism-related organs, whether capable of modulating inflammatory responses or regulating active molecular signals, are fundamental elements in the process of obesity, which would provide an environment factor as the cause of the complex pathology of obesity. There is compelling evidence supporting modulation of microbiota to treat obesity and related disorders.

Dietary intake appears to be a major regulator of the structure and function of gut microbiota. Results show that carbohydrate restriction and diets rich in fiber and vegetables are associated with health benefits due in part to microbial changes (Cotillard et al., 2013; Mardinoglu et al., 2018). Administration of prebiotics, probiotics and synbiotics have long been proposed as ways of modifying metabolic disorders, which are largely dependent upon altered microbiota composition. Multi-strain probiotic supplementation can reduce liver transaminases, tumor necrosis factor-α level and insulin resistance (Sepideh et al., 2016). Additionally, probiotic *Lactobacillus rhamnosus* GG is effective in the prevention of hepatic steatosis and injury partly through modulation of hepatic AMPK activation (Zhang et al., 2015), and probiotic strain *Bifidobacterium animalis subsp*. *Lactis 420* supplementation reduces bacterial translocation of Gram-negative bacteria from the *Enterobacteriaceae* group to normalize adipose-tissue inflammation (Amar et al., 2011). Interventions with prebiotics can also modulate gut microbiota and significantly reduce body weight, percent body fat, and desire for high-calorie foods, as well as improve insulin sensitivity, low-grade chronic inflammation and lipid metabolism (Dewulf et al., 2013; Hume et al., 2017; Nicolucci et al., 2017). In addition to its effect on peripheral organs, prebiotic supplementation also improves appetite control in children with obesity (Hume et al., 2017).

A rather harsh method of modulating microbial composition is FMT, which can alter the entire microbial community. FMT is a way to normalize the composition and functionality of gut microbiota by transferring an infusion of a fecal suspension from a healthy individual to the gastrointestinal tract of another person. This method has now become widely accepted as a highly successful rescue treatment for recurrent *Clostridium difficile* infection (Drekonja et al., 2015). Related data concerning FMT as a treatment for obesity and related metabolic disorders in humans are relatively sparse. Transplanting fecal matter from lean donors into obese or individuals with metabolic syndromes was recently examined. Although the results indicated no significant decrease in BMI at 6-weeks post-transplantation, there was a significant increase in insulin sensitivity (Vrieze et al., 2012; Kootte et al., 2017). Additionally, loss of microbial diversity is common in patients with obesity, and gut-microbial diversity was increased significantly after FMT from a lean donor. Notably in this case, the number of butyrate-producing bacteria was increased; however, whether enhanced diversity or changes in specific bacterial species contribute to the effect of FMT remains unknown.

## CONCLUSION

Considering the key role of gut microbiota in host metabolism, mechanistic investigations of microbiota modulation have demonstrated its restorative potential for both gut-microbiota composition and functionality. Therefore, such modulation represents a promising strategy for compositional variations and a potential therapeutic target for the treatment of obesity and other metabolic diseases. However, there remains considerable controversy regarding the precise role of gut microbiota in obesity, and more interventional clinical trials are critical for continued progress.

## ABBREVIATIONS

ACC: acetyl CoA carboxylase
AMPK: adenosine monophosphate activated protein kinase
BMI: body mass index
CCK: cholecystokinin
ChREBP: carbohydrate responsive element binding protein
CNS: central nervous system
EC: enterochromaffin
FAS: fatty acid synthase
Fiaf: fasting-induced adiposity factor
FMT: fecal microbiota transplantation
FXR: farnesoid X receptor
GABA: γ-aminobutyric acid
GF: germ free
GI: gastrointestinal
GLP-1: glucagon like peptide 1
GPR: G-protein coupled receptor
HFD: high-fat diet
LPL: inhibiting lipoprotein lipase
LPS: lipopolysaccharides
NAFLD: non-alcoholic fatty liver disease
NASH: nonalcoholic steatohepatitis
NTS: nucleus tractus solitaries
PYY: peptide YY
SCFA: short-chain fatty acids
SREBP-1: sterol regulatory element-binding protein 1
TLRs: Toll-like receptors
